# Comprehensive Bioenergetic Evaluation of Microbial Pathway Variants in Syntrophic Propionate Oxidation

**DOI:** 10.1128/mSystems.00814-20

**Published:** 2020-12-08

**Authors:** Mauricio Patón, Héctor H. Hernández, Jorge Rodríguez

**Affiliations:** a Department of Chemical Engineering, Research and Innovation Center on CO_2_ and H_2_ (RICH), Khalifa University, Abu Dhabi, United Arab Emirates; b Department of Biomedical Engineering, Khalifa University, Abu Dhabi, United Arab Emirates; Marquette University

**Keywords:** bioenergetics modeling, metabolic pathway modeling, metabolic energy conservation, interspecies electron transfer

## Abstract

In this work, a systematic methodology was developed (based on known biochemistry, physiology, and bioenergetics) for the automated feasibility evaluation and net ATP yield quantification of large sets of pathway variants. Possible pathway variants differ in their intermediate metabolites, in which electron carriers are involved, in which steps are consuming/producing ATP, and in which steps are coupled to (and to how many) proton (or its equivalent) translocations. A pathway variant is deemed feasible, under a given set of physiological and environmental conditions, only if all pathway reaction steps have nonpositive Gibbs energy changes and if all the metabolite concentrations remain within an acceptable physiological range (10^−6^ to 10^−2^ M). The complete understanding of syntrophic propionate oxidation remains elusive due to uncertainties in pathways and the mechanisms for interspecies electron transfer (IET). Several million combinations of pathway variants and parameters/conditions were evaluated for propionate oxidation, providing unprecedented mechanistic insight into its biochemical and bioenergetic landscape. Our results show that, under a scenario of optimum environmental conditions for propionate oxidation, the *Smithella* pathway yields the most ATP and the methylmalonyl-coenzyme A (CoA) pathways can generate sufficient ATP for growth only under a cyclical pathway configuration with pyruvate. The results under conditions typical of methanogenic environments show that propionate oxidation via the lactate and via the hydroxypropionyl-CoA pathways yield the most ATP. IET between propionate oxidizers and methanogens can proceed either by dissolved hydrogen via the *Smithella* pathway or by different mechanisms (e.g., formate or direct IET) if other pathways are used.

**IMPORTANCE** In this work, an original methodology was developed that quantifies bioenergetically and physiologically feasible net ATP yields for large numbers of microbial metabolic pathways and their variants under different conditions. All variants are evaluated, which ensures global optimality in finding the pathway variant(s) leading to the highest ATP yield. The methodology is designed to be especially relevant to hypothesize on which microbial pathway variants should be most favored in microbial ecosystems under high selective pressure for efficient metabolic energy conservation. Syntrophic microbial oxidation of propionate to acetate has an extremely small quantity of available energy and requires an extremely high metabolic efficiency to sustain life. Our results bring mechanistic insights into the optimum pathway variants, other metabolic bottlenecks, and the impact of environmental conditions on the ATP yields. Additionally, our results conclude that, as previously reported, under specific conditions, IET mechanisms other than hydrogen must exist to simultaneously sustain the growth of both propionate oxidizers and hydrogenotrophic methanogens.

## Abstract (precis)

In this work, an original methodology was developed that quantifies bioenergetically and physiologically feasible net ATP yields for large numbers of microbial metabolic pathways and their variants under different conditions. All variants are evaluated, which ensures global optimality in finding the pathway variant(s) leading to the highest ATP yield.

## INTRODUCTION

Propionate oxidation to acetate and hydrogen is a highly endergonic reaction under standard conditions (Δ*G*^01^ = +76.1 kJ/mol propionate [1]). The reaction, however, can become exergonic and yield sufficient energy for net ATP production only at very low hydrogen partial pressures (P_H2_) (1 to 10 Pa) typically found in methanogenic environments (2–4). Although the reduction reaction of CO_2_ to methane via hydrogen is highly exergonic under standard conditions (Δ*G*^01^ = −131 kJ/mol [1]), under typical methanogenic conditions (very low P_H2_) the reaction falls much closer to equilibrium, with actual quantities of energy available between −15 and −40 kJ/mol (3). Volatile fatty acid (VFA) oxidizers and methanogens are both known to grow very close to thermodynamic equilibrium (5).

Due to these bioenergetic limitations, propionate oxidation is believed to occur primarily under syntrophic association with hydrogen-scavenging microorganisms (6). The specialized nature of methanogenic archaea, such as hydrogenotrophic methanogens, which are able to grow only on a very few substrates (hydrogen and/or formate) (4, 7), makes them dependent on other microorganisms for their supply of substrate. Both syntrophic reactions can proceed simultaneously within a narrow range of concentrations if dissolved hydrogen is the interspecies electron transfer (IET) mechanism. This range is known as the methanogenic niche. The fact that, under methanogenic conditions, the amount of energy available from either of the two syntrophic reactions is smaller than the minimum needed for one ATP unit of synthesis via substrate-level phosphorylation (SLP) implies that metabolic energy conservation must be driven by chemiosmotic transmembrane proton (or its equivalent, such as sodium or potassium) translocations (1).

Numerous studies have focused on elucidating the catabolic pathways of propionate oxidation to acetate, for which numerous different possible pathways have been described, including (i) propionate oxidation via the methylmalonyl-coenzyme A (CoA) pathway, which has been extensively studied (4, 6, 8–16), (ii) propionate oxidation via lactate (12, 17, 18), or (iii) propionate oxidation via hydroxypropionyl-CoA (12, 17). Propionate oxidizers that use the methylmalonyl-CoA pathway are, however, the only ones that have been isolated (6). Propionate oxidation via an alternative butyrate- and acetate-yielding pathway has also been reported (19, 20).

In addition, significant work has been done on the elucidation of electron transfer mechanisms between syntrophic partners, propionate (or butyrate) oxidizers with methanogens. Different mechanisms for IET have been proposed to occur via hydrogen and/or formate. Although IET via hydrogen has been identified as more suitable than formate due to its higher diffusivity (21), IET via formate has also been proposed when microorganisms do not grow in aggregates, given its much higher solubility (6, 8, 9, 22, 23). Formate and hydrogen production in the same microorganism have also been proposed to take place at different reaction sites, with (i) formate produced at the reoxidation step of menaquinone from the oxidation of succinate to fumarate and (ii) hydrogen produced in the reoxidation of the NADH from the malate oxidation to oxaloacetate and the ferredoxin reduction of pyruvate to acetyl-CoA reactions, respectively (4, 10). The hypothesis of both IET-capable species being simultaneously produced is supported by faster observed growth in the presence of syntrophic methanogens that metabolize both hydrogen and formate (24, 25). Formate has been suggested to serve in those cases as a temporary electron sink (11).

Although detailed thermodynamic studies have been conducted on individual reactions present in related microbial catabolic pathways (26–31), the complete understanding of many microbial conversions remains unachieved. This is largely due to the lack of clarity on the different possible pathway variants and/or mechanisms that drive endergonic reactions. Pathway variants in this work consist of any possible configurations compatible with known biochemistry and physiology and are defined in terms of which intermediate metabolites (including which specific electron carriers) are involved and in terms of the mechanisms and locations in which energy conservation by proton translocations or SLP take place within the pathway. A comprehensive bioenergetic evaluation of a very large set of pathway variants is presented in this work for propionate oxidation as well as for its syntrophic counterpart, hydrogenotrophic methanogenesis. The impact that intermediate metabolite concentrations have on the bioenergetics of the reaction step is central to determine the feasibility of each pathway variant and the quantification of its net ATP yield. The syntrophic pathway evaluation for an ample range of hydrogen partial pressures is specifically targeted to understand the limits of the IET mechanism and of the methanogenic niche within which syntrophic propionate oxidizers and methanogens both can simultaneously sustain growth.

## RESULTS AND DISCUSSION

The pathway variants with the highest ATP yield, along with their corresponding metabolite concentration profiles and proton translocation configurations, are presented and discussed.

### Propionate oxidation.

**FIG 1:**
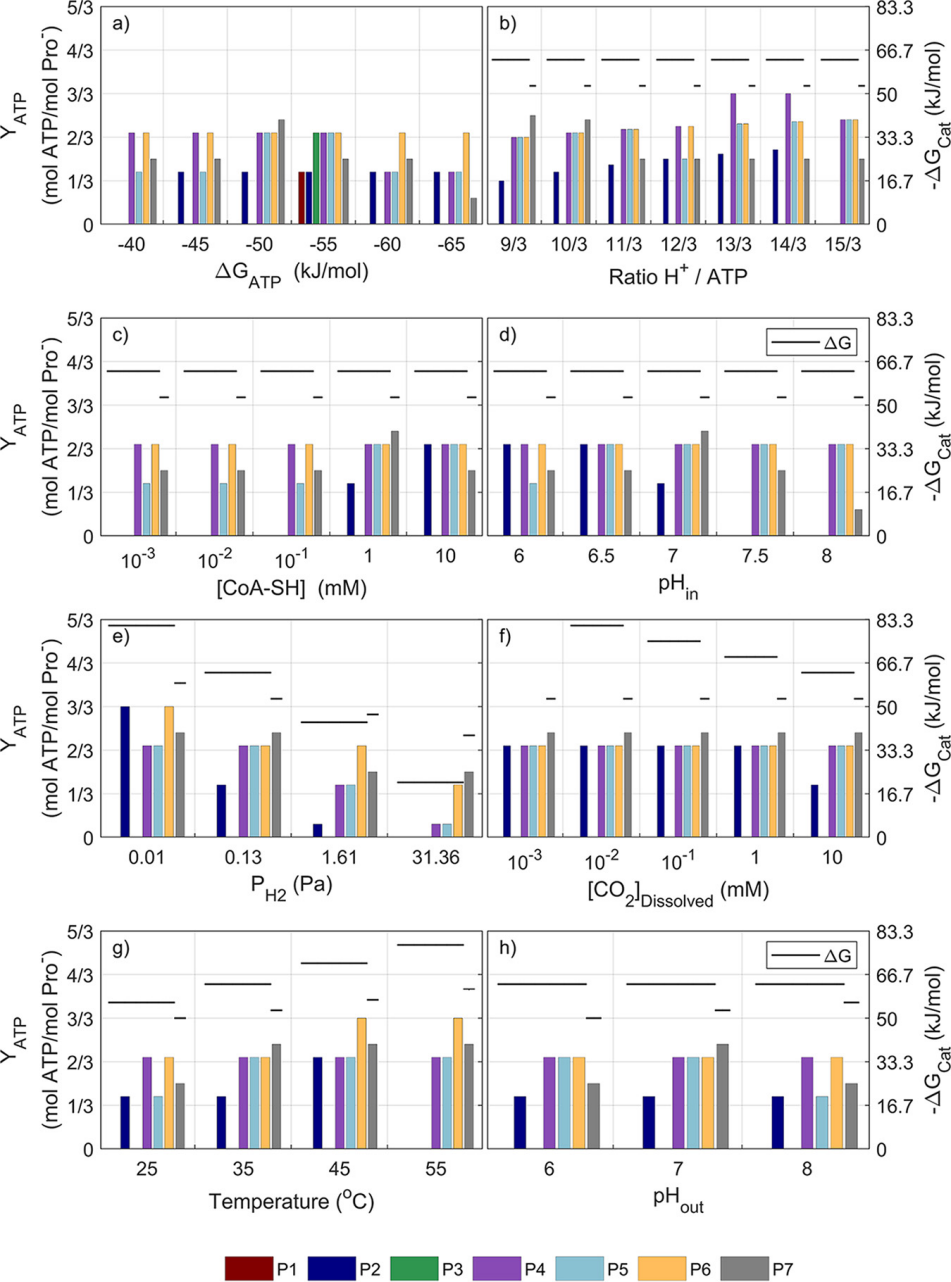
Net ATP produced for each pathway under different physiological (a to d) and environmental (e to h) parameter values is shown for the optimal concentrations for propionate degraders (scenario *Opt*). Only the parameter indicated below each graph is modified with respect to the reference conditions (Table 3). The horizontal dark lines showing the total catabolic energy available in each pathway (−Δ*G*_cat_) allow for pathway efficiency visualization.

**FIG 2:**
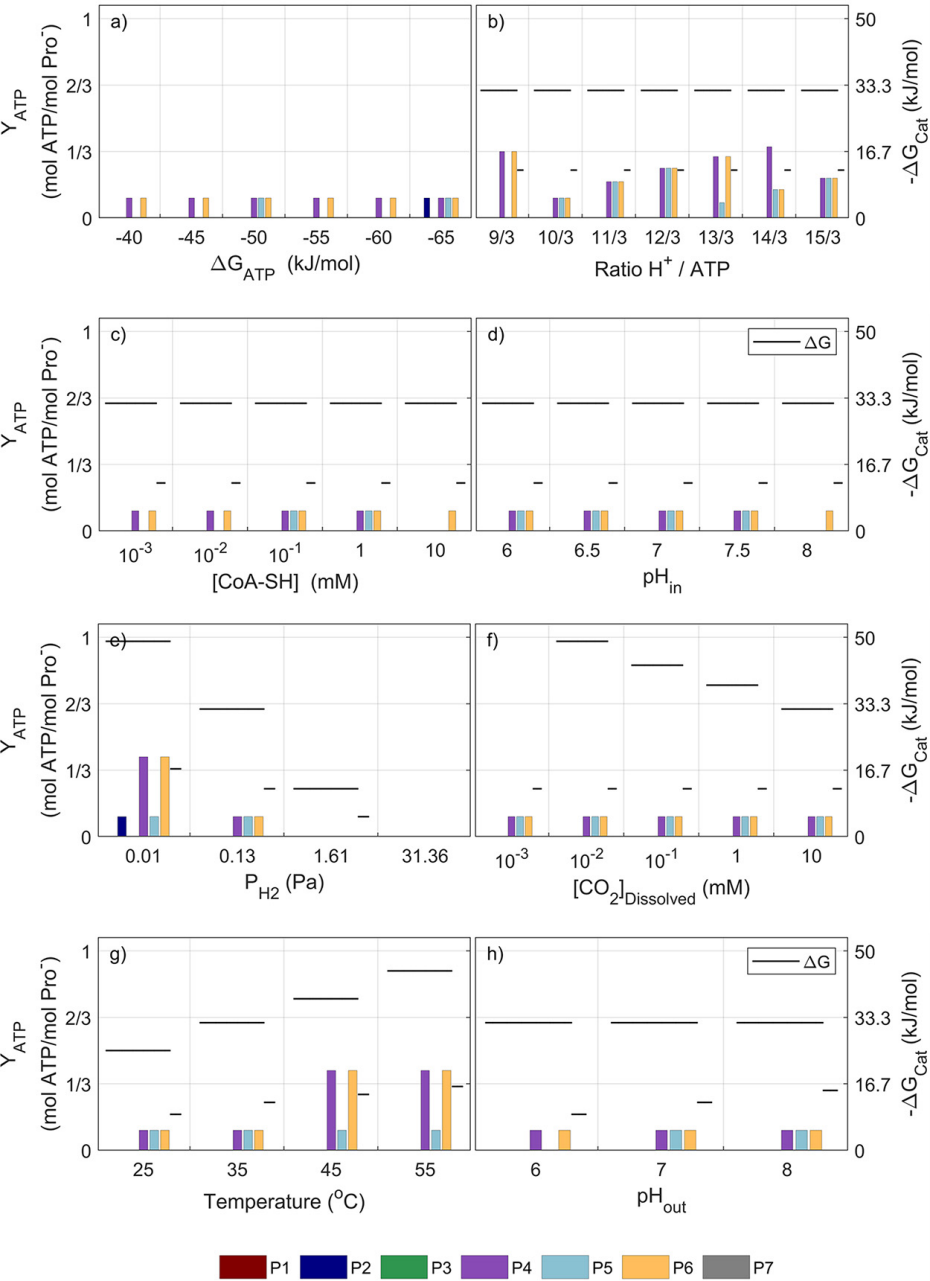
Net ATP produced for each pathway under different physiological (a to d) and environmental (e to h) parameter values is shown for a typical methanogenic environment (scenario *Met*). Only the parameter indicated below each graph is modified with respect to the reference conditions (Table 3). The horizontal dark lines showing the total catabolic energy available in each pathway (−Δ*G*_cat_) allow for pathway efficiency visualization.

Figures 1 and 2 present (for scenarios *Opt* and *Met*, respectively [see Materials and Methods]) the results of the positive net ATP yield of the feasible propionate oxidation pathways for different values of the physiological parameters and environmental conditions around the default values (see Table 3). The net ATP yields presented are the maxima found for each pathway considering all possible combinations of electron carriers and all possible configurations for proton translocations (Tables 1 and 2).

**TABLE 1:**
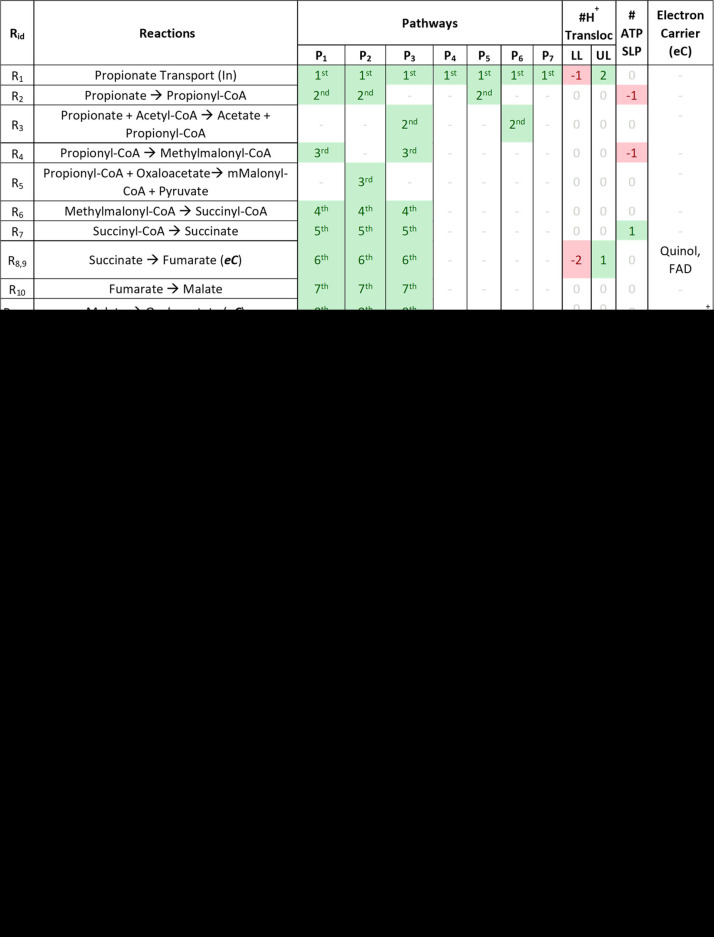
The complete set of pathway reactions considered for propionate oxidation to acetate per equations 1 and 2^*a*^

a The reactions considered for electron carrier regeneration are also included (eC_Reg_). The numbers under the pathways (P_n_) indicate the order in which reactions occur in each pathway. Lower (LL) and upper (UL) limits for the number of proton translocations in a specific reaction step and ATP consumption/production as the substrate-level phosphorylation (SLP) for each reaction are indicated.

**TABLE 2:**
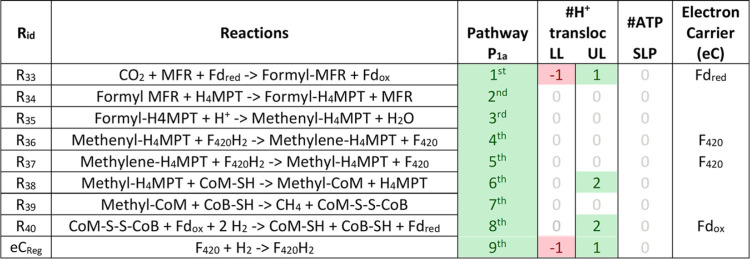
The set of reactions considered for CO_2_ reduction with H_2_ to methane^*a*^

a The reactions considered for electron carrier regeneration are also included (eC_Reg_). The numbers under the pathways (P_n_) indicate the order in which reactions occur in each pathway. Lower (LL) and upper (UL) limits for the number of proton translocations in a specific step are indicated. No ATP consumption/production via substrate-level phosphorylation (SLP) was reported for this reaction.

Results under the scenario *Opt* (Fig. 1a) show that, at the default value of −50 kJ/mol of Δ*G*_ATP_, the pathway of Smithella propionica (P_7_) is the one yielding the most net ATP. It appears that a higher or lower Δ*G*_ATP_ value would lead to lower efficiency of the P_7_ pathway. The lactate and hydroxypropionyl-CoA pathways appear to be those capable of producing the most ATP for the oxidation of propionate under the stoichiometry of equation 1. The methylmalonyl-CoA pathway (arguably the most frequently reported for propionate oxidation) appears to be feasible in most cases only via the cyclical configuration with pyruvate (R_5_ in Table 1). This is in agreement with several literature observations (9, 11, 16). Alternative configurations for the methylmalonyl-CoA pathway (P_1_ and P_3_) appear feasible only for Δ*G*_ATP_ values of −55 kJ/mol.The value of the H^+^/ATP ratio (Fig. 1b) shows that small energy quanta (up to an optimum H^+^/ATP ratio of 13/3) could favor the efficiency of the propionate oxidation pathways (equation 1) (P_1–6_) with highly efficient energy conservation from the total available in the lactate pathway (see Fig. S5 in the supplemental material).Conversely, the *Smithella* pathway (P_7_) appears to yield diminishing net ATP production when the quantum of energy becomes smaller. The concentration values of free CoA (Fig. 1c) do not appear to impact the efficiency of the lactate pathway in the range of values evaluated, but they show an optimum range (1 to 10 mM) for the methylmalonyl-CoA (P_2_) and the *Smithella* (P_7_) pathways. Values for intracellular pH (Fig. 1d) appear to show an optimum for the methylmalonyl-CoA (P_2_) and the *Smithella* (P_7_) pathways at a neutral pH, while the rest of the configurations (P_4–6_) appear to be unaffected by pH. The detailed effect of the intracellular pH on the lactate pathway can be seen in Fig. S6.

Regarding the differences in the environmental conditions considered (Table 3), it is worth noting that these imply changes in the overall catabolic energy available. Lower values of P_H2_ (Fig. 1e) make the overall reaction more exergonic, and potentially more net ATP can be produced. Due to the different amounts of hydrogen produced in the two propionate oxidation stoichiometries (equations 1 and 2), their total catabolic energies available are different and are impacted differently by P_H2_, even intersecting at some values. At a P_H2_ higher than 30 Pa, the *Smithella* pathway (P_7_) remains the most exergonic with respect to the other pathways (P_1–6_) and is favored for higher net ATP yield production (Fig. 1e).

**TABLE 3:** Set of physiological parameters and environmental conditions evaluated for propionate oxidizers and hydrogenotrophic methanogens^a^

Parameter	Unit	Value						
Physiological								
ΔG_ATP_	kJ/mol	−40	−45	**−50**^(a)(b)^	−55	−60	−65	
H^+^/ATP		**9/3**^(b)^	**10/3**^(a)^	11/3	12/3	13/3	14/3	15/3
CoA-SH	mM	10^−3^	10^−2^	10^−1^	**1**^(a)^	10		
CoM-SH	mM	10^−3^	10^−2^	10^−1^	**1**^(b)^	10		
H_4_MPT	mM	10^−3^	10^−2^	10^−1^	**1**^(b)^	10		
pH_in_		6	6.5	**7**^(a)(b)^	7.5	8		
Environmental conditions								
H_2_	Pa	0.01	**0.13**^(a)^	1.62	**31.63**^(b)^	316.3		
CO_2_	mM	10^−3^	10^−2^	10^−1^	1	**10**^(a)(b)^		
T^*b*^	^o^C	**25**^(b)^	**35**^(a)^	45	55			
pH_out_		6	**7**^(a)(b)^	8				

a The values indicated were evaluated for each parameter individually, leaving all other parameters at the default reference value (in boldface). Label (a) refers to default values for propionate oxidizers, and label (b) refers to default values for hydrogenotrophic methanogens. CoA-SH and CoM-SH refer to the concentrations of their free forms.

b For hydrogenotrophic methanogenesis, a sensitivity analysis was not performed for temperature. All ATP yields are computed with enthalpies at 25°C.

Analogously, for the dissolved external CO_2_ concentration (also a product of the overall reaction), the lower its concentration, the more catabolic energy is available for pathways P_1–6_. However, under the environmental conditions of scenario *Opt*, the net ATP yield remains unaffected for all pathways. It appears that even at high CO_2_ concentrations the carboxylation step (R_4_) does not proceed; therefore, methylmalonyl-CoA pathways (P_1_ and P_3_) do not yield any net ATP. For propionate oxidation via methylmalonyl-CoA to be feasible, the configuration needs to involve no carboxylations, as is the case in the cyclical configuration (P_2_ and R_5_).

The impact of temperature (Fig. 1g) can be observed in terms of higher temperature leading to more exergonic overall reactions (due to increases in entropy under these stoichiometries), allowing for more pathway variants to obtain positive and higher net ATP yields.

The extracellular pH (Fig. 1h) shows almost no impact due to the very similar acidity (pK_a_ values) for propionate and acetate (substrate and product of the overall reaction) (equation 1), while it seems to affect the *Smithella* pathway (P_7_) due to the impact on the bioenergetics of the reaction of the protons (which is one of the products of equation 2).

Results under the scenario *Met* (Fig. 2) correspond to typical methanogenic conditions under which much lower energy is available for microbial growth than under the previous optimum conditions of the scenario *Opt*. Therefore, lower net ATP yields are observed for every pathway under scenario *Met*. Under these conditions, the *Smithella* pathway reaction (equation 2) does not have sufficient available energy to translocate one proton and yields no net ATP.

The results consistently present the lactate and the hydroxypropionyl-CoA pathways (P_4_ and P_6_) as the ones biochemically and thermodynamically capable of yielding the most ATP from propionate oxidation under the methanogenic conditions. Only for Δ*G*_ATP_ values of −65 kJ/mol (Fig. 2a) does it appear that the oxidation via methylmalonyl-CoA (P_2_) could yield a similar net ATP.

The H^+^/ATP ratio (Fig. 2b) shows that a small energy quantum (up to an optimum H^+^/ATP ratio of 14/3) could favor the efficiency of the lactate pathway. The concentration of free CoA (Fig. 2c) does not appear to impact the efficiency of the lactate pathway in the range of values evaluated. The net ATP yields by the lactate and the hydroxypropionyl-CoA pathway (P_4–6_) appear to be unaffected by intracellular pH within the range covered.

The different environmental conditions considered (Table 3) show tendencies similar to those in the scenario *Opt*. Lower values for P_H2_ (Fig. 2e) make the overall reaction more exergonic, and potentially more net ATP can be produced. However, even at very low P_H2_ values (1.62 Pa), such as those found in methanogenic environments, the energy available is below the energy threshold for one net proton translocation. As in scenario *Opt*, CO_2_ concentration does not impact the net ATP yield for the pathways considered (Fig. 2f).

Analogously to scenario *Opt*, the impact of temperature in scenario *Met* (Fig. 2g) can be observed in terms of higher temperature leading to a more exergonic overall reaction (due to increases in entropy under these stoichiometries), allowing for a higher net ATP yield for the lactate and hydroxypropionyl-CoA pathways (P_4,6_).

The extracellular pH (Fig. 2h) shows almost no impact due to the very similar acidity (pK_a_ values) for propionate and acetate (substrate and product of the overall reaction).

**FIG 3:**
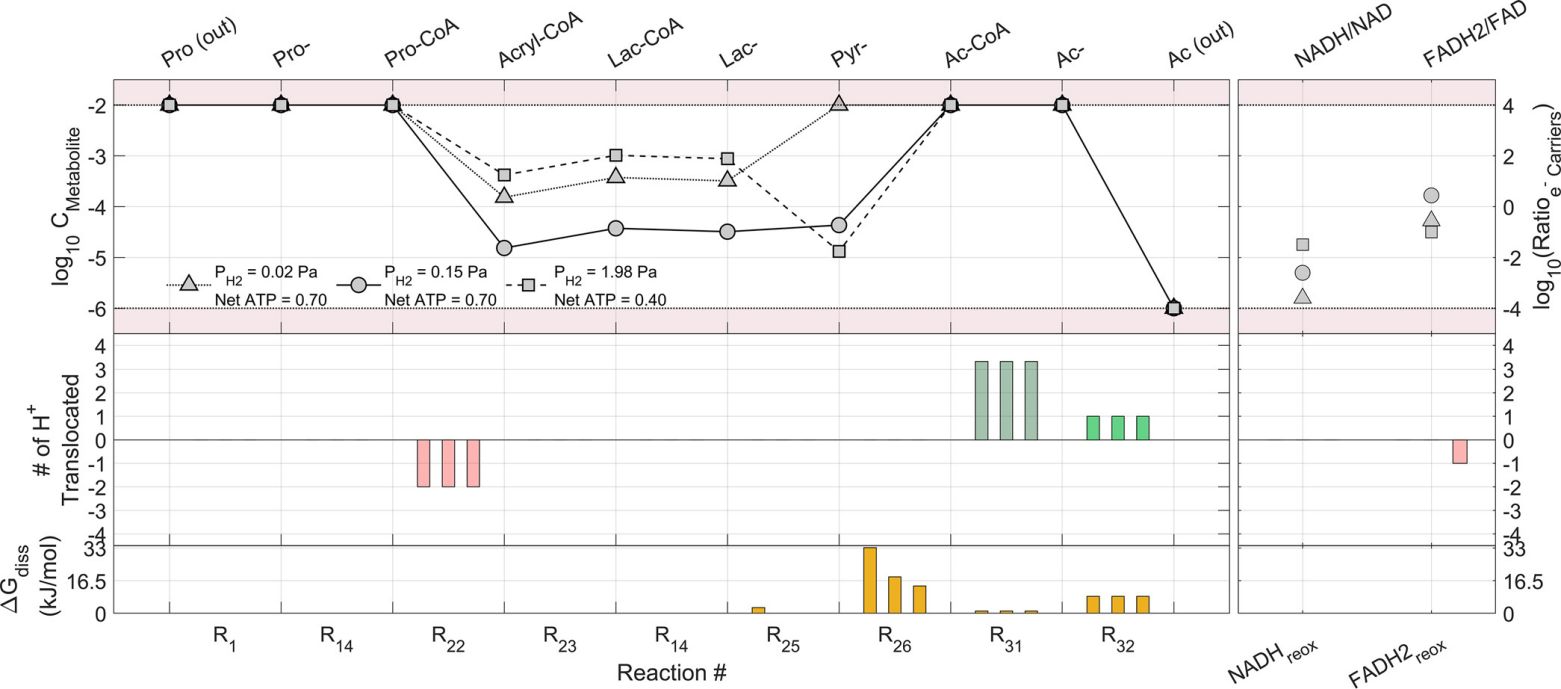
Pathway metabolite concentrations in the propionate oxidation pathway via lactate (P_4_) at different hydrogen partial pressures (P_H2_). Symbols in gray (top) indicate the logarithmic concentration of each metabolite as labeled in the upper axis. Concentrations outside the physiological limits fall in the shaded red area. Green and red bars (middle) indicate energy conservation reactions in which either energy is recovered or consumed to fuel a reaction via proton translocations. Darker green bars indicate ATP production via substrate-level phosphorylation. Yellow bars (bottom) indicate Gibbs free energy dissipations (losses) at that step in the pathway. The default physiological parameters and environmental conditions from Table 3 were used (other than that for P_H2_).

To enable detailed pathway and bottleneck analyses, the intermediate metabolite concentration profiles of all feasible reactions are provided by the automated method developed for the pathway evaluation. In Fig. 3, the profile is shown for the pathway variants that appeared to yield the most net ATP, namely, propionate oxidation via lactate (P_4_), at three different partial pressures of hydrogen (P_H2_).

Figure 3 shows how all metabolites remain within physiological limits for all the P_H2_ values evaluated. As the catabolic energy decreases with increasing product concentration (P_H2_), less net energy in the form of translocated protons can be recovered by the cell, particularly in the reoxidation of FADH_2_. For those reactions with products potentially exceeding the maximum physiological concentrations thermodynamically (e.g., pyruvate to acetyl-CoA), energy is dissipated (as described in Materials and Methods). Figure 3 also clearly illustrates the energetic bottlenecks of the lactate pathway (steps leading to very low product concentrations), namely, (i) the oxidation of propionyl-CoA to acryloyl-CoA, for which the influx of two protons is needed, and (ii) the conversion of lactate to pyruvate, highly sensitive to the P_H2_ value.

### Hydrogenotrophic methanogenesis.

**FIG 4:**
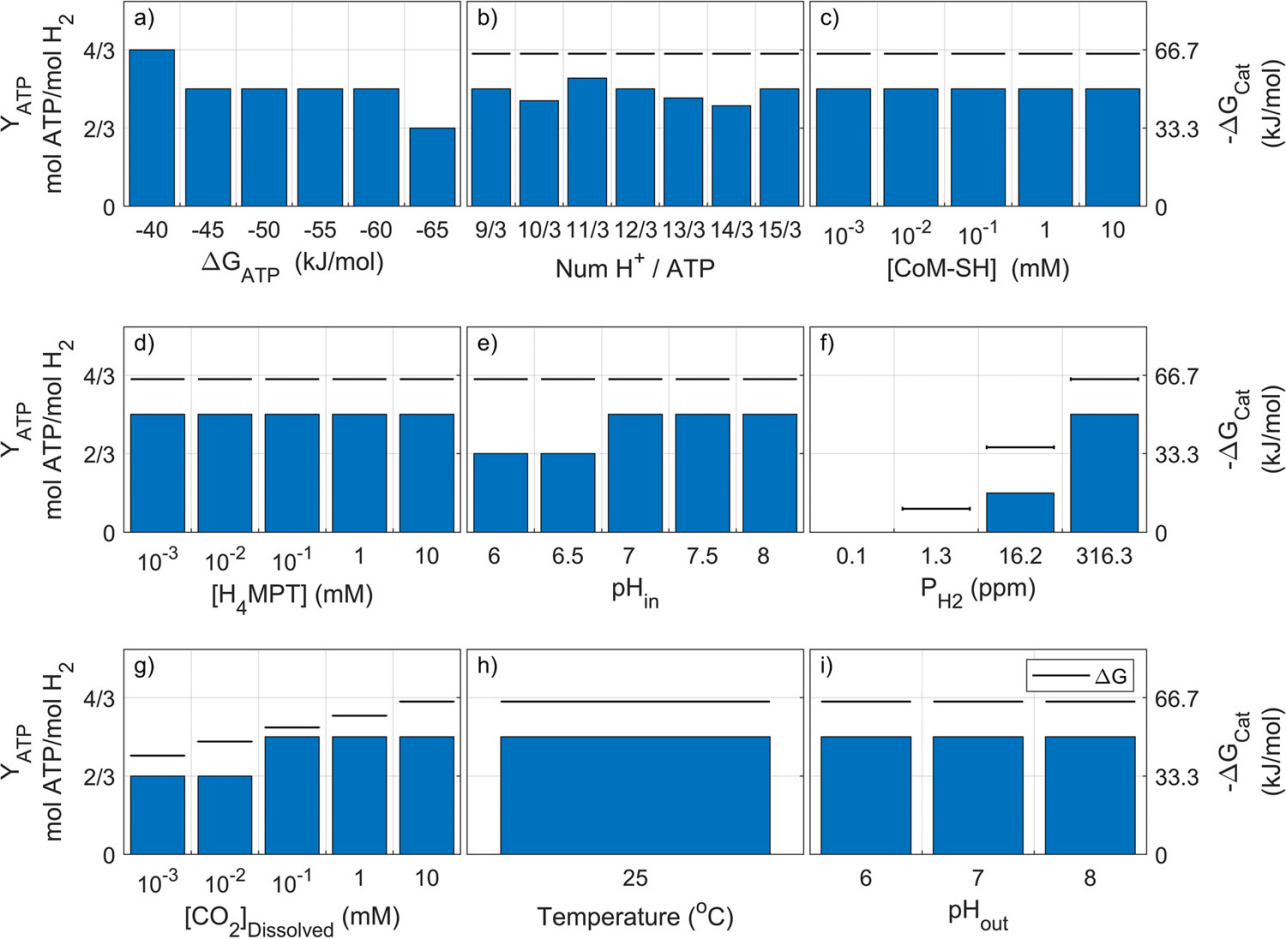
Net ATP equivalents produced in the hydrogenotrophic methanogenesis pathway for different physiological parameters (a to e) and environmental conditions (f to i). In each plot only one parameter, as indicated, is modified with respect to the default conditions from Table 3. Temperature (h) could only be evaluated at 25°C due to unavailable enthalpies of formation data for several key components present in the pathway.

In Fig. 4, the results obtained from the evaluation of the hydrogenotrophic methanogenesis pathway are presented in terms of net ATP yield as a function of different physiological parameters and environmental conditions around the default values in Table 3.

During hydrogenotrophic methanogenesis, no ATP is produced by substrate-level phosphorylation, and the value of Δ*G*_ATP_ only impacts the size of the energy quantum in reactions with proton translocation. As shown in Fig. 4, the reaction has no sensitivity to Δ*G*_ATP_ in the range from −45 to −60 kJ/mol, while an energy quantum of 40 kJ/mol or smaller appears to allow for one additional net proton translocation. At the default reference Δ*G*_ATP_, the optimum H^+^/ATP ratio appears to be 11/3. Intracellular pH values lower than 7 appear to decrease the net ATP, while no effect is shown from the concentrations of CoM or H_4_MPT within the evaluated ranges.

As for the case of propionate oxidation, different environmental conditions imply differences in the overall catabolic energy available (with the exception of extracellular pH, since no net acidity was produced or consumed). Since hydrogen and CO_2_ (Fig. 4f and g) are substrates of the hydrogenotrophic methanogenesis reaction, the higher their concentration the higher the catabolic energy available and, potentially, the higher the net ATP recovered.

### Syntrophic propionate oxidation and methanogenesis: methanogenic niche.

**FIG 5:**
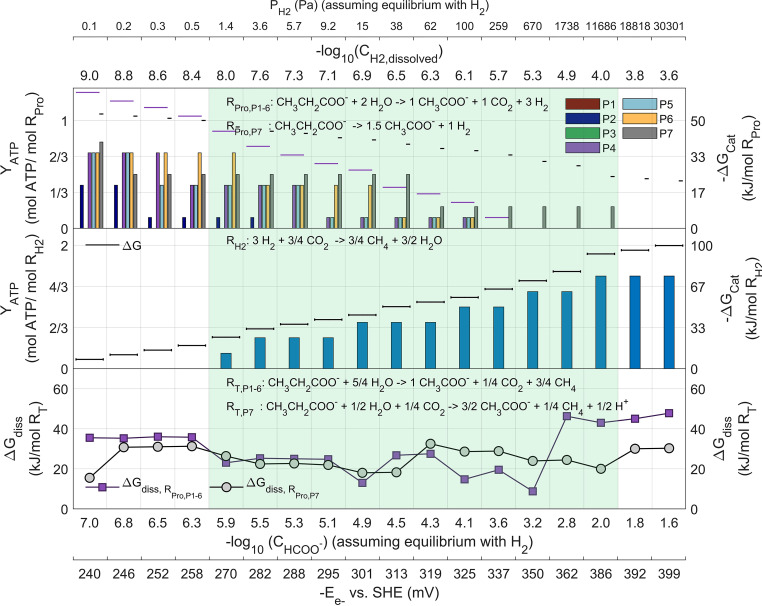
ATP yields under optimal conditions for propionate degraders (scenario *Opt*) for propionate oxidizers (top) and hydrogenotrophic methanogens (at 25°C; middle) as functions of P_H2_ are shown in bars corresponding to the feasible reactions with positive net ATP yields. Horizontal lines indicate the available catabolic energy, in purple for propionate oxidation to acetate (P_1–6_) and in black for the *Smithella* pathway (P_7_). A range of hydrogen partial pressures is shown where both microbial functional groups (a propionate degrader and a hydrogenotrophic methanogen) could sustain growth and coexist (shaded green area). The bottom plot shows the total energy dissipated (lost) in each complete syntrophic reaction. Additional axis for alternative IET via formate and direct electron transfer shows their values of concentration and voltage equivalent (in equilibrium) with the hydrogen concentrations and pressures shown.

**FIG 6:**
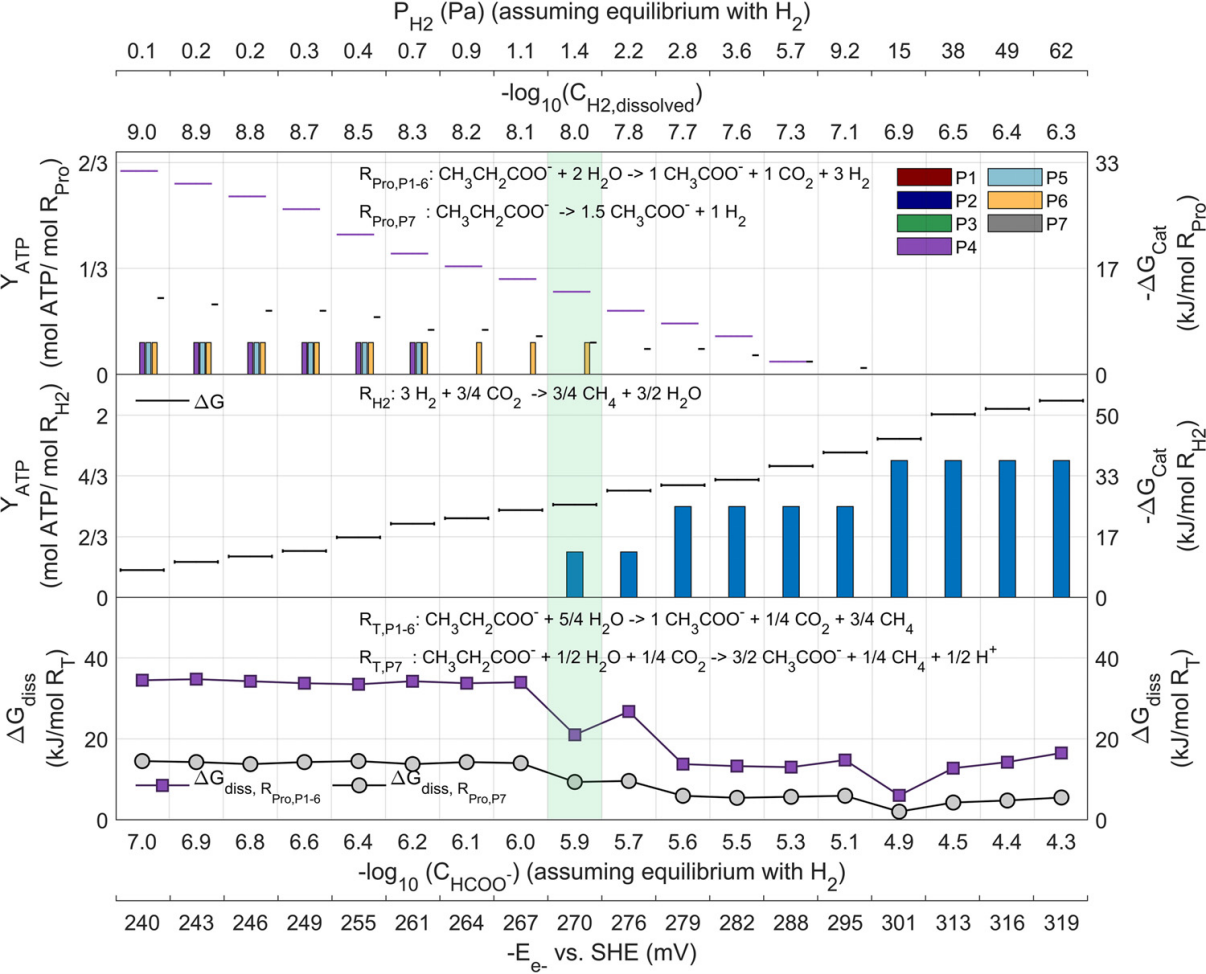
ATP yields under methanogenic conditions (scenario *Met*) for propionate oxidizers (top) and hydrogenotrophic methanogens (at 25°C; middle) as functions of P_H2_ are shown in bars corresponding to the feasible reactions with positive net ATP yields. Horizontal lines indicate the available catabolic energy, in purple for propionate oxidation to acetate (R_P1–6_) and in black for the *Smithella* pathway (R_P7_). A range of hydrogen partial pressures is shown where both microbial functional groups (a propionate degrader and a hydrogenotrophic methanogen) could sustain growth and coexist (shaded green area). The bottom plot shows the total energy dissipated (lost) in each complete syntrophic reaction. Additional axis for alternative IET via formate and direct electron transfer shows their values of concentration and voltage equivalent (in equilibrium) with the hydrogen concentrations and pressures shown.

The pathway evaluation method developed was also applied to gain insight into the syntrophic growth of propionate oxidizers and hydrogenotrophic methanogens. The maximum net ATP yield achievable by each of the two microbial groups was evaluated as a function of the dissolved hydrogen concentration (or its corresponding partial pressure), widely accepted as the syntrophic link for interspecies electron transfer (IET). The energetically equivalent values for alternative possible IET mechanisms are also shown, namely, the concentration of formate and the electron potential for any possible direct IET. The values shown for both are in thermodynamic equilibrium with the corresponding P_H2_. All other default parameters as per Table 3 were used for both microbial groups. The methanogenic niche was evaluated under the two environmental conditions as previously defined, namely, scenario *Opt* (Fig. 5) and scenario *Met* (Fig. 6).

Although in scenario *Opt* the conditions for propionate oxidation are optimal, propionate oxidizers following the pathways P_1–6_ (stoichiometry as per equation 1) show a limited syntrophic coexistence range of P_H2_ (or equivalent alternative IET) between 1.2 and 100 Pa. A complete evaluation of all possible pathway variant feasibilities shows that, within this syntrophic P_H2_ range, propionate oxidation can generate net ATP only via the lactate or the hydroxypropionyl-CoA pathway (P_4–6_). The methylmalonyl-CoA pathway (P_2_) was shown to be able to generate only net ATP at P_H2_ of 3.6 Pa or below.

The very low values of P_H2_ for syntrophic growth coexistence correspond to dissolved hydrogen concentrations between 10^−8^ and 10^−6.1^ M, below the defined minimum physiological limit of 10^−6^ M. This poses a fundamental problem if we consider that, for a bacterial cell volume of circa 1 μm^3^, the number of hydrogen molecules present inside a cell within this concentration range would be as few as 6 to 480. Such small numbers imply a kinetic impossibility for methanogenesis to actually occur. This supports the idea that IET between syntrophic partners should occur through alternative or additional mechanisms other that via dissolved hydrogen. Sustained growth for the methanogenic syntrophic partner, if based solely on dissolved hydrogen as the electron donor, appears theoretically impossible according to this analysis.

The equivalent concentrations of formate (taken as thermodynamic equilibrium with hydrogen) are shown in Fig. 5. An alternative formate IET mechanism appears feasible with concentrations above the defined lower physiological limit (1 μM). Observations of highly expressed enzymes for the reoxidation of quinone or ferredoxin that produce formate in propionate oxidizers such as *Pelotomaculum* (16) are in support of formate as an IET mechanism. Direct electron transfer via conductive materials at potentials between −270 and −325 mV also appears feasible (Fig. 5).

The syntrophic growth coexistence P_H2_ range if the propionate oxidation takes place via the *Smithella* pathway (P_7_) is, however, much wider, reaching feasible P_H2_ values of up to 11,000 Pa. This corresponds to dissolved hydrogen concentrations up to 10^−4^ M, well within physiological limits.

These results indicate that the IET mechanism for syntrophic propionate oxidation under the stoichiometry from equation 1 is infeasible via dissolved hydrogen and feasible via formate or a direct electron transfer alternative. At the same time, the results indicate that dissolved hydrogen is a feasible IET mechanism if the propionate oxidation takes place via the *Smithella* stoichiometry and pathway (P_7_).

The syntrophic coexistence niche was also evaluated under the typically observed conditions in methanogenic environments of scenario *Met* (less favorable for propionate oxidation). The results under these conditions are shown in Fig. 6.

Interestingly, under scenario *Met*, the conditions are so restricted energetically that the net ATP from all pathways is substantially lower. As opposed to the scenario *Opt*, shown in Fig. 5, the syntrophic coexistence of the P_H2_ range becomes even narrower, and the *Smithella* pathway (P_7_) does not even seem to be possible.

Propionate oxidation seems possible only via hydroxypropionyl-CoA and syntropy via IET, unlike dissolved hydrogen. The *Smithella* pathway (P_7_) never makes any net ATP feasible due to the catabolic energy available being lower than the minimum required for single-proton translocation.

These dramatically different results between the conditions of the two scenarios suggest that the actual concentrations encountered under local conditions (e.g., by microorganisms growing within aggregates, such as a granules) must fall between both or have a spatial variability and differ substantially from those measured in the bulk liquid of anaerobic digestion reactors.

### Conclusions.

The automated pathway analysis method developed in this work provides an unprecedented capability to evaluate large numbers of pathway configurations. This allows for the evaluation of any known, and even postulated, biochemistry to theoretically determine the feasible pathways (physiologically and thermodynamically) with the highest ATP yield. The method, entirely mechanistic and largely founded on first principles, brings insights for the study of energy-limited microbial metabolisms.

Propionate oxidation was evaluated in the entire domain of possible pathway variations within the known biochemistry and the thermodynamic and physiological feasibility, applied to all reaction steps and all metabolite concentrations. Under a scenario of optimum environmental conditions, the oxidation of propionate via the *Smithella* pathway yields the most ATP, and the methylmalonyl-CoA pathways can generate sufficient ATP for growth only under a cyclical pathway configuration with pyruvate (P_2_). Under a scenario of typical methanogenic conditions, the oxidation of propionate via the lactate and via the hydroxypropionyl-CoA pathways appears to yield the most ATP.

Extremely low P_H2_ values (below the minimum reasonable physiological limits) appear to be required to sustain syntrophic growth coexistence with methanogens if propionate is oxidized to acetate and three hydrogens (P_1–6_), while this is not observed for the *Smithella* stoichiometry and pathways (P_7_). This implies that IET via dissolved hydrogen is not feasible under pathways P_1–6_ and must occur via alternative mechanisms that could include formate or direct electron transfer (e.g., via conductive pili). Conversely, dissolved hydrogen appears a feasible IET if the propionate oxidation goes via the *Smithella* pathway (P_7_). The very different results predicted under most favorable or methanogenic typical conditions suggest that local concentrations or spatial variability via microbial aggregates must be occurring to explain the literature observations for syntrophic propionate oxidation.

## MATERIALS AND METHODS

### Selection of pathways for propionate oxidation.

The selection of the possible catabolic pathways for the oxidation of propionate to acetate with the overall stoichiometry shown in equation 1 was compiled from a comprehensive literature review (4, 6, 9, 10, 12, 14, 17). The oxidation of propionate to acetate via butyrate through the alternative pathway proposed for Smithella propionica per equation 2 was also included (19, 20).

(1) $${\text{R}}_{\text{Pro,P1–6}}{\text{: CH}}_{\text{3}}{\text{CH}}_{\text{2}}{\text{COO}}^{-}{\;+\text{ 2 H}}_{\text{2}}\text{O }\overset{}{⇄}{\text{ CH}}_{\text{3}}{\text{COO}}^{-}\;+{\text{ CO}}_{\text{2}}\;+{\text{ 3 H}}_{\text{2}}$$

(2) $${\text{R}}_{\text{Pro,P7}}{\text{: CH}}_{\text{3}}{\text{CH}}_{\text{2}}{\text{COO}}^{\text{−}}\;+{\text{ H}}_{\text{2}}\text{O }\overset{}{⇄}{\text{ 3/2 CH}}_{\text{3}}{\text{COO}}^{\text{−}}\;+\text{ 1}{\text{ H}}_{2}\;+\;1/2{\text{ H}}^{+}$$

Diversity was found in the literature concerning what electron carriers are involved in specific reaction steps of the methylmalonyl-CoA pathway. In the oxidation of succinate to fumarate, menaquinone has been reported as the electron carrier (9, 11), while FADH_2_ has also been reported as a possible electron carrier for the same reaction step (13). Discrepancies in the specific terminal products from electron carrier reoxidation were also found. The oxidation of NADH carriers has been proposed to occur through hydrogenases (11, 32–34). Formate dehydrogenases have also been reported to oxidize menaquinone (9, 16). Hydrogen and formate, however, appear to be thermodynamically equivalent (10, 35); therefore, only hydrogen was considered in this work as the terminal product of the electron carrier oxidations.

The pathway steps for hydrogenotrophic methanogenesis were also obtained from the literature (2, 3, 7, 36–38), specifically including the energy conservation sites via proton translocation (39, 40).

Selected pathway reactions were cross referenced from the literature sources and the Kyoto Encyclopedia of Genes and Genomes (KEGG) database (41). Only reactions based on enzymes reported in prokaryotes were considered. Propionate oxidation via the methylmalonyl-CoA pathways (P_1_ to P_3_ in Table 1) corresponds to pathways described for microorganisms such as Syntrophobacter wolinii, Pelotomaculum schinkii, or P. propionicicum (4, 9, 11, 42). Propionate oxidation via butyrate (P_7_ in Table 1) corresponds to the pathway described for Smithella propionica (20, 43). Propionate oxidation via lactate (P_4_ in Table 1) corresponds to the pathway in the opposite direction as described (17). Propionate oxidation via hydroxypropionyl-CoA (P_5–6_ in Table 1) corresponds to a compilation of previously proposed pathways (12) and possible reactions found in KEGG that could occur in microorganisms that may not have been isolated yet (e.g., P_5_ or P_6_ in Table 1). In this work, all reactions in a given pathway variant were assumed to occur within a single cell. It is worth noting that some of the pathways selected, such as methylmalonyl-CoA (P_2_ and P_3_), lactate (P_4_), and hydroxypropionyl-CoA (P_6_), contain cyclic steps (4, 11, 14). The complete set of pathways considered for the oxidation of propionate is presented in Table 1 and those for hydrogenotrophic methanogenesis in Table 2. Graphical representations of the pathways are available in Fig. S1 in the supplemental material.

10.1128/mSystems.00814-20.5 FIG S1 Schematic representations of the pathways selected for propionate oxidation, summarized in Table 1. Download FIG S1, TIF file, 0.6 MB. Copyright © 2020 Patón et al. 2020 Patón et al. This content is distributed under the terms of the Creative Commons Attribution 4.0 International license.

### Environmental conditions for pathway evaluation.

The propionate oxidation pathways were evaluated under two different scenarios (namely, *Opt* and *Met*). Under scenario *Opt*, the most favorable environmental concentrations for a propionate oxidizer, i.e., the maximum permitted concentration (10^−2^ M) for propionate and minimum concentration (10^−6^ M) for acetate, were selected. Scenario *Opt* somehow assumes that those extremely favorable local concentrations could occur at some point for cells living, e.g., in aggregates (that can differ significantly from the ones measured in the anaerobic bulk environment). Under scenario *Met*, environmental conditions similar to those found in a typical stable methanogenic anaerobic digester under steady-state operation were selected (44). The propionate and acetate concentration values were set at 1.4·10^−4^ M and 3.07·10^−3^ M, respectively. The hydrogen partial pressure (P_H2_) was set to a default of 1.62 Pa, assumed to be in equilibrium with its corresponding dissolved concentration. The evaluation of all pathway variants is conducted for each scenario independently under the indicated constant extracellular concentrations of substrates and products.

### Intracellular metabolite concentrations.

Based on the values for intracellular metabolite concentrations reported in the literature (45) and on theoretical calculations (46), all internal metabolite concentrations were constrained within a physiologically feasible maximum of 10^−2^ M and a minimum of 10^−6^ M. The small volume of a cell (circa 1 μm^3^) (47) implies that fewer than 100 single molecules would be present inside the cell at 10^−7^ M, a number considered too low for any feasible subsequent positive reaction rate. The total concentrations of other conserved moieties, such as electron carriers and free CoA, were defined as parameters (29, 48). The concentrations of electron carriers were determined by the ratios between their reduced and oxidized forms and constrained such that their total concentration is conserved and neither form falls outside the above physiological limits (this implies maximum and minimum reduced/oxidized carrier ratios of 10^−4^ and 10^4^, respectively). All these assumptions were made due to the lack of data regarding the concentrations of internal metabolites for propionate oxidizers. Experimental measurements of such concentrations would provide certainty in some of the assumptions followed and help in providing a better estimation of the pathways evaluated.

### Thermodynamic parameters and assumptions.

The thermodynamic values of Gibbs free energy (and enthalpies) of formation, required for the thermodynamic calculations for each reaction step, were collected from the literature for each metabolite (1, 3, 49–52). The enthalpy of formation values of a few metabolites involved in the oxidation of propionate to acetate are unavailable in the literature and had to be estimated. Detailed references for the thermodynamic parameters, along with the estimation methods used for some enthalpies, are provided in the Text S1 and Data Set S1. Temperature-corrected bioenergetics were applied to all pathway reactions for propionate oxidation using the Van’t Hoff equation. In the case of the pathway reactions for hydrogenotrophic methanogenesis, temperature corrections could not be applied due to the unavailability of enthalpies for methanofuran (MFR), tetrahydromethanopterin (H_4_MPT), or its related components (methyl-MFR). However, the evaluation of the hydrogenotrophic methanogenesis corresponds to the most exergonic scenario, as higher temperatures decrease the full catabolic energy available for the hydrogenotrophic methanogen reaction. Therefore, the evaluation of the pathway at 25°C provides an upper bound of the ATP that can be produced by this group of microorganisms.

10.1128/mSystems.00814-20.1 TEXT S1 Information describing the algorithms used in Materials and Methods and the estimation of enthalpies of components. It also includes additional figures that support the Discussion. Download Text S1, DOCX file, 0.1 MB. Copyright © 2020 Patón et al. 2020 Patón et al. This content is distributed under the terms of the Creative Commons Attribution 4.0 International license.

10.1128/mSystems.00814-20.2 DATA SET S1 A spreadsheet that describes the calculation of the enthalpies of the components. In addition, the values estimated and used for the evaluation of the pathways can be checked in Table S1. Download Data Set S1, XLSX file, 0.02 MB. Copyright © 2020 Patón et al. 2020 Patón et al. This content is distributed under the terms of the Creative Commons Attribution 4.0 International license.

### Chemiosmotic energy conservation.

All reactions identified to take place via membrane-bound enzymes were assumed to be capable of proton translocation through the cell membrane, either to directly recover energy as a proton-motive force (pmf) or to drive endergonic reactions in a pathway. Those energy conservation sites were identified both through previous literature (1, 4, 39, 53–58) and the online database Metacyc (59).

### Assessment of pathway feasibility.

For each reaction step in which an electron carrier was involved, a set of possible electron carrier variants was defined. Additionally, for each reaction step with proton translocation capability, a range of possible numbers of proton translocations that can take place in that step were defined (Table 1).

All possible variants, combinatorial of all electron carrier variations with all possible numbers of proton translocations in the capable sites, were evaluated for each pathway. The feasibility of any given pathway variant is evaluated by seeking a zero or minimum Gibbs energy dissipation in all pathway reaction steps. This corresponds to minimum energy dissipation and maximum catabolism efficiency of the pathway. This criterion allows for the sequential calculation of the subsequent product concentrations at each pathway step given that of the substrate from the previous step.

The evaluation of a pathway variant consists first of the determination of its feasibility. A pathway is feasible only if all reaction steps have a zero or negative Gibbs energy change and all intermediate metabolite concentrations can still remain within the physiological limits. A pathway variant is deemed unfeasible and is discarded if any of the metabolite concentrations must fall below the lower physiological limit in order to thermodynamically enable a preceding reaction to occur. In the opposite case, if a reaction step is highly exergonic and allows for the produced metabolite to take concentration values higher than the upper physiological limit (10^−2^ M) while still showing Δ*G* of <0, then the concentration must sit at the upper physiological limit and energy must dissipate and be lost. The evaluation of a pathway variant that is feasible concludes with the quantification of its overall net ATP yield.

Some of the pathways evaluated contain cycles (e.g., P_2_ to P_4_ from Table 1). A pathway contains a cycle when one reaction in the pathway requires two substrates to yield two products (excluding the conserved moieties, such as electron carriers and free CoA). A specific section of the pathway evaluation algorithm was developed to evaluate the cyclic steps and metabolite concentrations based on the exact same principles described above and without the need for additional assumptions (Fig. S2 and S3).

10.1128/mSystems.00814-20.6 FIG S2 Algorithm used to solve pathways that contain loop reactions. Download FIG S2, TIF file, 0.4 MB. Copyright © 2020 Patón et al. 2020 Patón et al. This content is distributed under the terms of the Creative Commons Attribution 4.0 International license.

10.1128/mSystems.00814-20.7 FIG S3 Algorithms used to solve branched pathways and the algorithm used to solve the electron bifurcation reaction for hydrogenotrophic methanogenesis. Download FIG S3, TIF file, 0.3 MB. Copyright © 2020 Patón et al. 2020 Patón et al. This content is distributed under the terms of the Creative Commons Attribution 4.0 International license.

In addition to cycles, pathways can contain electron bifurcation reactions (58, 60), as is the case in the reduction of the CoM-CoB heterodisulfide in the methanogenesis pathway (R_34_ in Table 2). This allows for the reduction of CO_2_ to formyl-MFR via the produced reduced ferredoxin (R_26_ in Table 2). A specific section of the algorithm was also developed to evaluate pathways in which electron bifurcation takes place (Fig. S4).

10.1128/mSystems.00814-20.8 FIG S4 Pathway metabolite concentrations in the propionate oxidation via the lactate pathway (P4a) at different ratios of H^+^/ATP. Symbols in gray (top) indicate the logarithmic concentration of the metabolite as labeled in the upper axis. Concentration range outside the physiological limits is the shaded red area. Green and red bars (middle) indicate energy conservation reactions in which either energy is recovered or consumed to fuel a reaction via proton translocations. Darker green bar indicates a reaction in which ATP is produced by substrate-level phosphorylation. Yellow bars (bottom) indicate Gibbs free energy dissipation (loss) at each reaction step in the pathway. The physiological parameters and environmental conditions (other than for the H^+^/ATP ratio), set as a reference in Table 3, were used. Download FIG S4, TIF file, 0.4 MB. Copyright © 2020 Patón et al. 2020 Patón et al. This content is distributed under the terms of the Creative Commons Attribution 4.0 International license.

10.1128/mSystems.00814-20.9 FIG S5 Pathway metabolite concentrations in the propionate oxidation via lactate pathway (P4a) at different pH values. Symbols in gray (top) indicate the logarithmic concentration of the metabolite as labeled in the upper axis. Concentration range outside the physiological limits is the shaded red area. Green and red bars (middle) indicate energy conservation reactions in which either energy is recovered or consumed to fuel a reaction via proton translocations. Darker green bar indicates a reaction in which ATP is produced by substrate-level phosphorylation. Yellow bars (bottom) indicate Gibbs free energy dissipation (loss) at each reaction step in the pathway. The physiological parameters and environmental conditions (other than for the pH values), set as a reference in Table 3, were used. Download FIG S5, TIF file, 0.4 MB. Copyright © 2020 Patón et al. 2020 Patón et al. This content is distributed under the terms of the Creative Commons Attribution 4.0 International license.

10.1128/mSystems.00814-20.10 FIG S6 Pathway metabolite concentrations in propionate oxidation via methylmalonyl-CoA pathway (P2) at different Δ*G*_ATP_ values. Symbols in gray (top) indicate the logarithmic concentration of the metabolite as labeled in the upper axis. Concentration range outside the physiological limits is the shaded red area. Green and red bars (middle) indicate energy conservation reactions in which energy is either recovered or consumed to fuel a reaction via proton translocations. Darker green bar indicates a reaction in which ATP is produced by substrate-level phosphorylation. Yellow bars (bottom) indicate Gibbs free energy dissipation (loss) at each reaction step in the pathway. The physiological parameters and environmental conditions (other than for the Δ*G*_ATP_ values), set as a reference in Table 3, were used. Download FIG S6, TIF file, 0.5 MB. Copyright © 2020 Patón et al. 2020 Patón et al. This content is distributed under the terms of the Creative Commons Attribution 4.0 International license.

The combinatory set of possible pathway variants as defined above becomes very large (nearly 80,000 in this case) for each set of physiological parameters and environmental conditions as defined in Table 3. The automation capabilities of the algorithm as developed allowed for the evaluation of the complete domain of all possible pathway variants. This ensures that the pathway variants with the highest ATP yields as found must be the global optima in terms of metabolic energy conservation. To the best of our knowledge, such a methodology has not been applied so far in the literature.

### Parameter selection and sensitivity analysis.

The values reported in literature for some of the required physiological parameters show differences (Table 3). Different values of Δ*G*_ATP_ hydrolysis under physiological conditions have been reported that range from as small as −45 or −50 kJ/mol (1) to −60 to −70 kJ/mol (4). The number of protons translocated per turn of the ATP synthase has been widely reported as 9 protons per turn, resulting in 3 ATPs (which leads to the widely accepted ratio of 3 protons per ATP). However, the number of protons required for a complete turn of the ATP synthase is known to vary based on the number of c-subunits able to translocate protons in the ATP synthase (61). This number has been reported to vary from 8 to 15 c-subunits (4, 61–65), equivalent to an H^+^/ATP ratio of 2.7 to 5 (66). A similar modeling approach was proposed for the total number of protons per mole of ATP (26). Under this approach, the number of protons per mole of ATP was defined as an integer number. In our approach, a fractional number of protons per ATP is proposed, based on the previously explained total number of protons required for a full turn on the ATP synthase, which results in the generation of 3 ATPs. It is worth noting that for a ratio of 15 protons per 3 ATPs and with a Δ*G*_ATP_ of −50 kJ/mol, the minimum quantum for metabolic energy conservation could be as low as −10 kJ/mol, in line with previously reported values for minimum energy required for microbial growth (5, 67). Such low-energy quanta could enable energy conservation in microorganisms growing on substrates that yield very low metabolic energy, such as propionate.

Intracellular free coenzyme A (CoA-SH) concentrations have been previously reported to be as high as 10 mM (26) and measured in a butyrate culture to vary between 100 and 200 μM (68). Due to these differences in values, the impact of the CoA-SH (for propionate oxidizers) and CoM-SH (for methanogens) on the net ATP yields of all pathways was specifically evaluated at different concentrations. Additionally, the impact of environmental variables, such as temperature and pH, on the bioenergetics was also evaluated. The parameter values for the physiological and environmental conditions considered are shown in Table 3.

A total of 32 parameter set configurations were evaluated for all the pathway variants (which corresponded to 2.5 million pathway variant-parameter set scenarios). Within this evaluation space and among the feasible pathway variants (i.e., those with all reactions with a Δ*G*_R_ of ≤0 plus all metabolites within physiological limits), only those combinations with a positive net ATP yield are presented and discussed. All other pathway variants are deemed either unfeasible or unable to sustain microbial growth under the given conditions.

10.1128/mSystems.00814-20.3 DATA SET S2 A spreadsheet with the simulation results for the scenario *Opt* (the most favorable scenario for propionate oxidizers). It provides an overview of the results obtained with the framework presented in the manuscript. Download Data Set S2, XLSX file, 5.2 MB. Copyright © 2020 Patón et al. 2020 Patón et al. This content is distributed under the terms of the Creative Commons Attribution 4.0 International license.

10.1128/mSystems.00814-20.4 TABLE S1 Name of the metabolites and the thermodynamic properties used. Estimated enthalpy values are shown in blue. Download Table S1, DOCX file, 0.02 MB. Copyright © 2020 Patón et al. 2020 Patón et al. This content is distributed under the terms of the Creative Commons Attribution 4.0 International license.
