# Supplementary figures and images for: Comprehensive Bioenergetic Evaluation of Microbial Pathway Variants in Syntrophic Propionate Oxidation
Source: mSystems. 2020 Dec 8;5(6):e00814-20. doi: 10.1128/mSystems.00814-20 (PMC7743110; doi:10.1128/mSystems.00814-20)

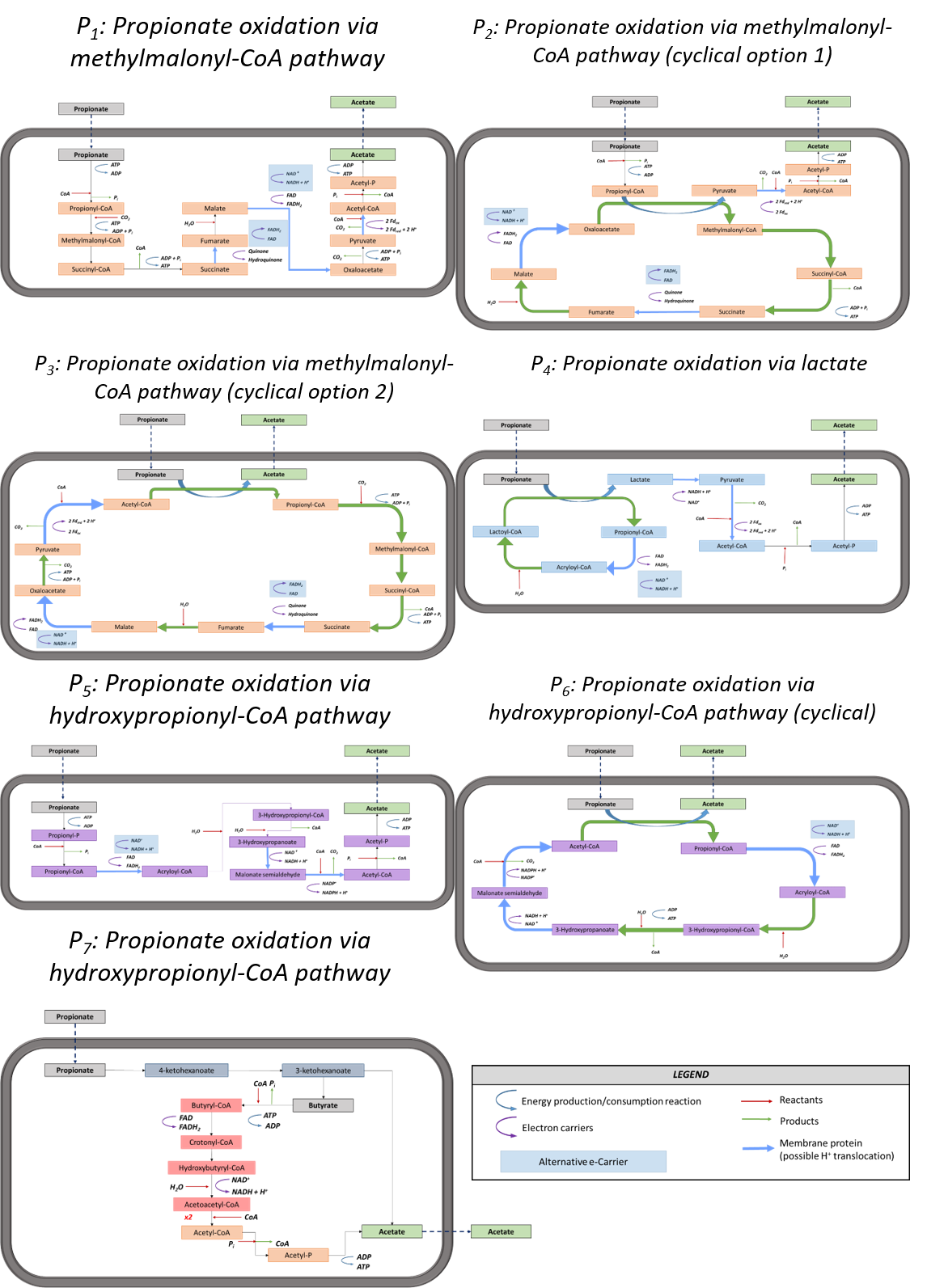

Supplement: FIG S1 [file mSystems.00814-20-sf001.tif]

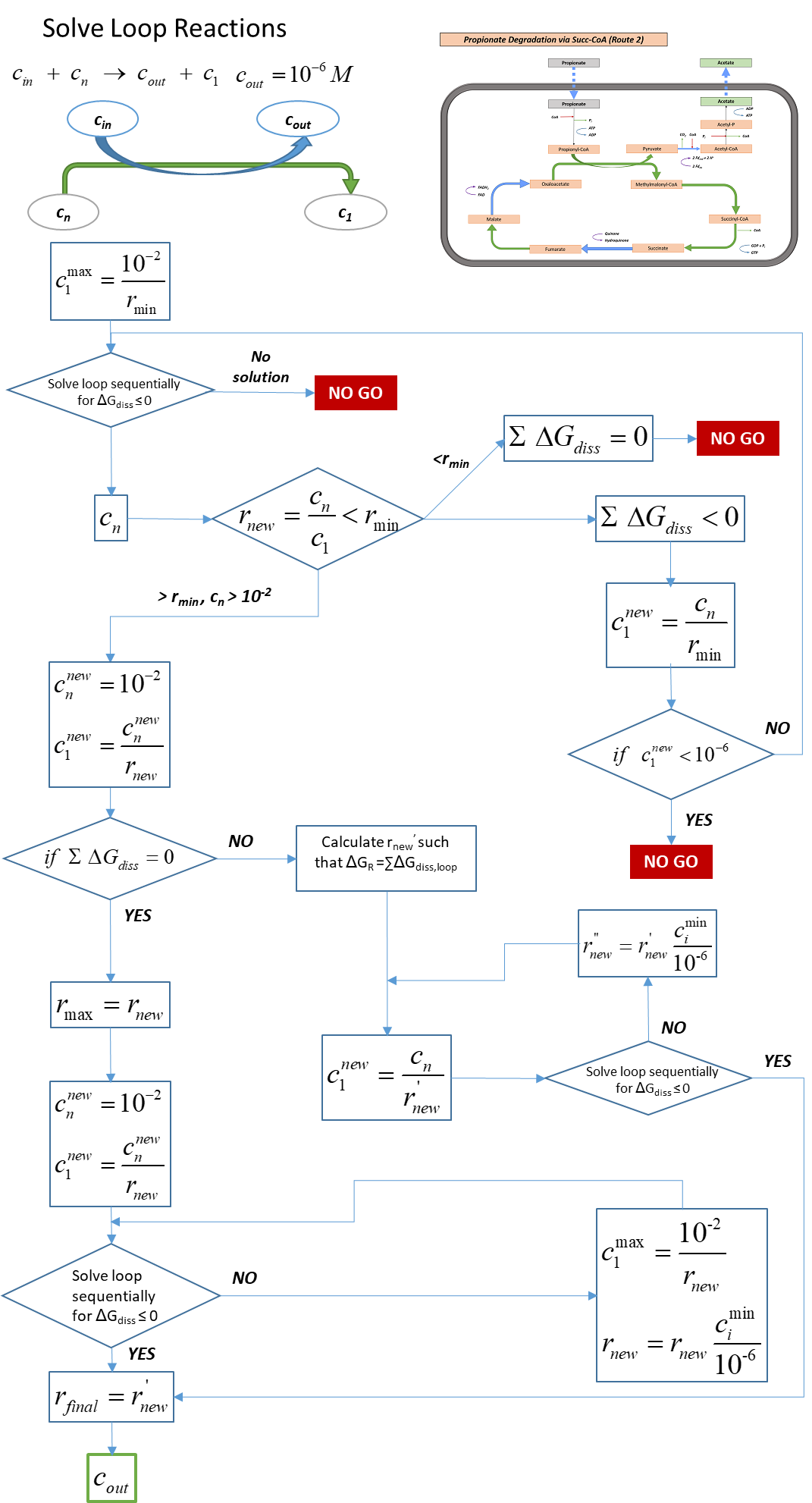

Supplement: FIG S2 [file mSystems.00814-20-sf002.tif]

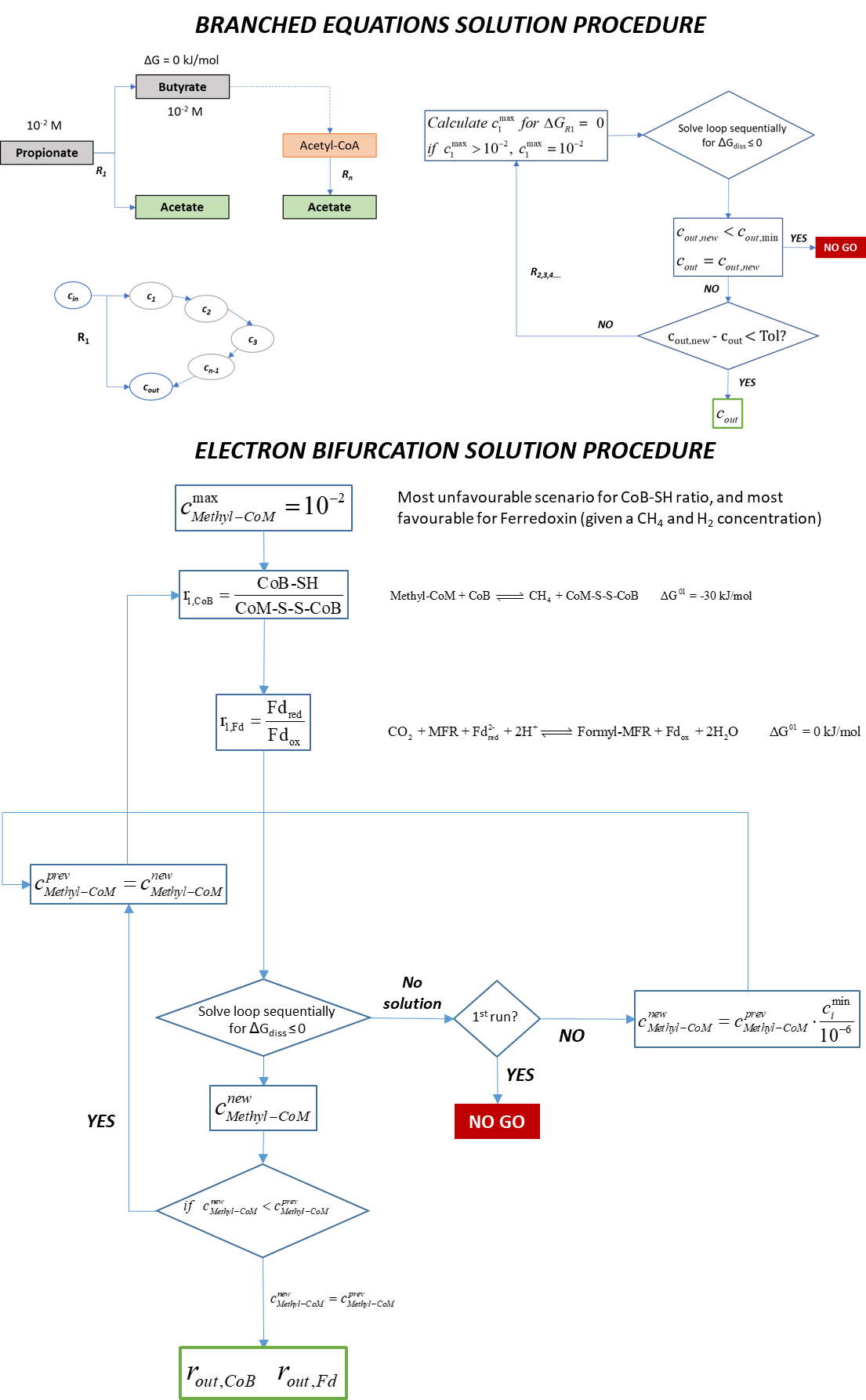

Supplement: FIG S3 [file mSystems.00814-20-sf003.tif]

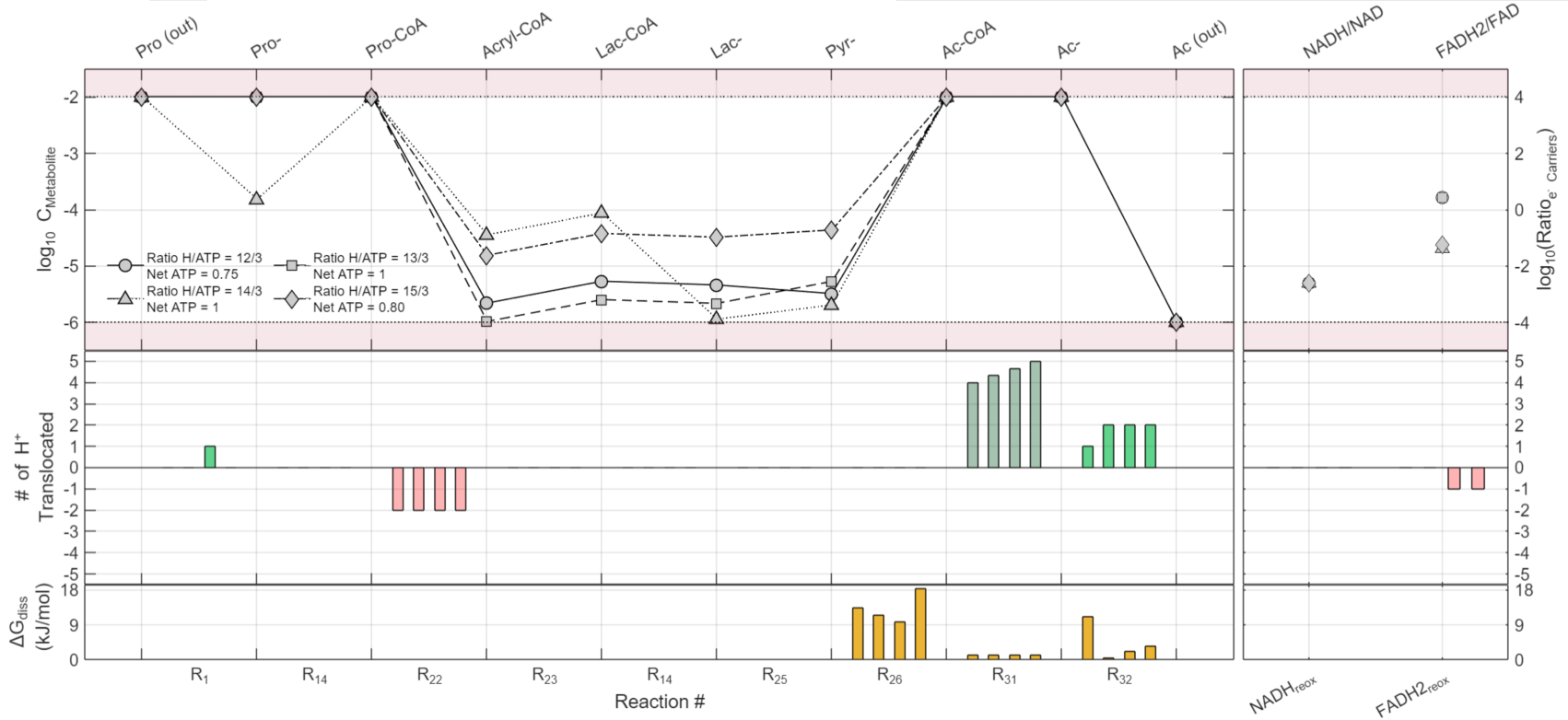

Supplement: FIG S4 [file mSystems.00814-20-sf004.tif]

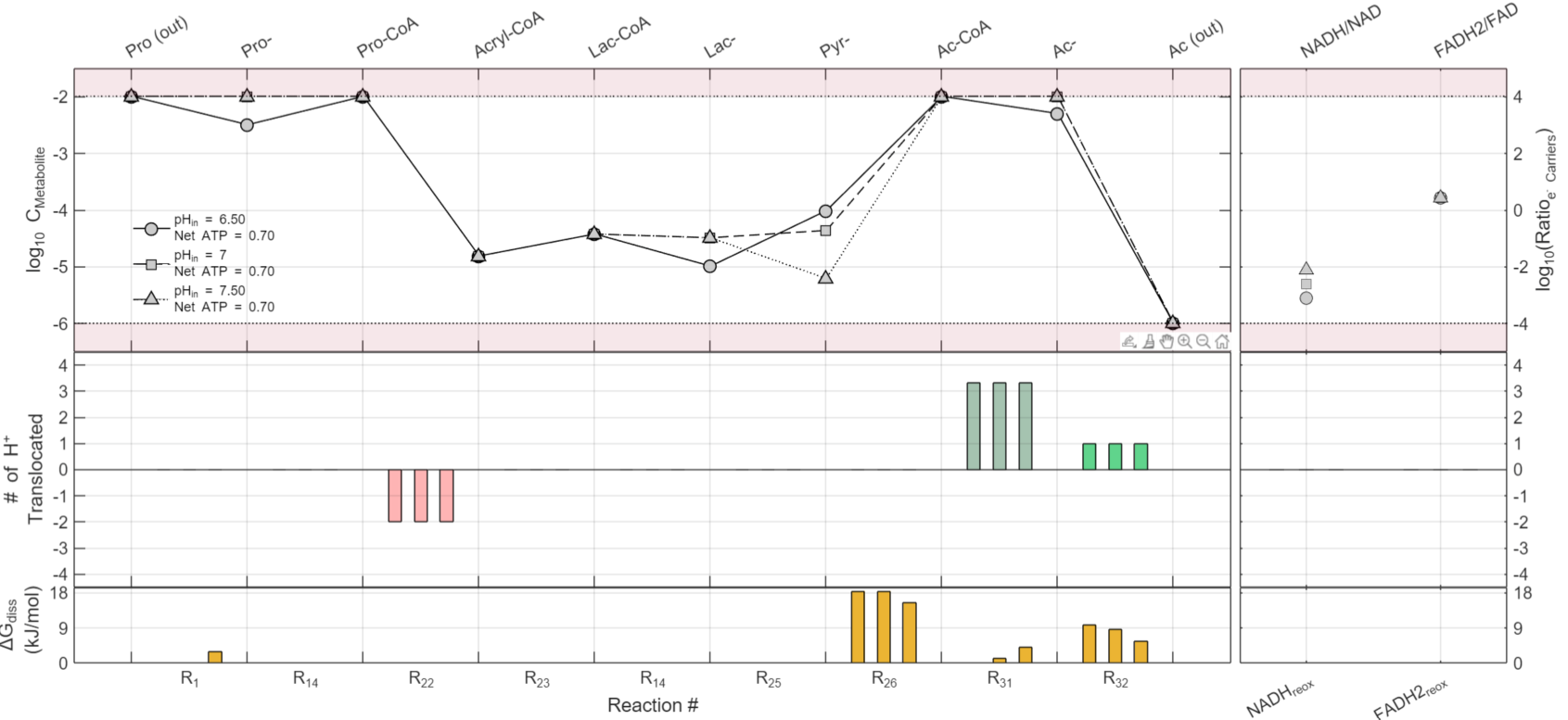

Supplement: FIG S5 [file mSystems.00814-20-sf005.tif]

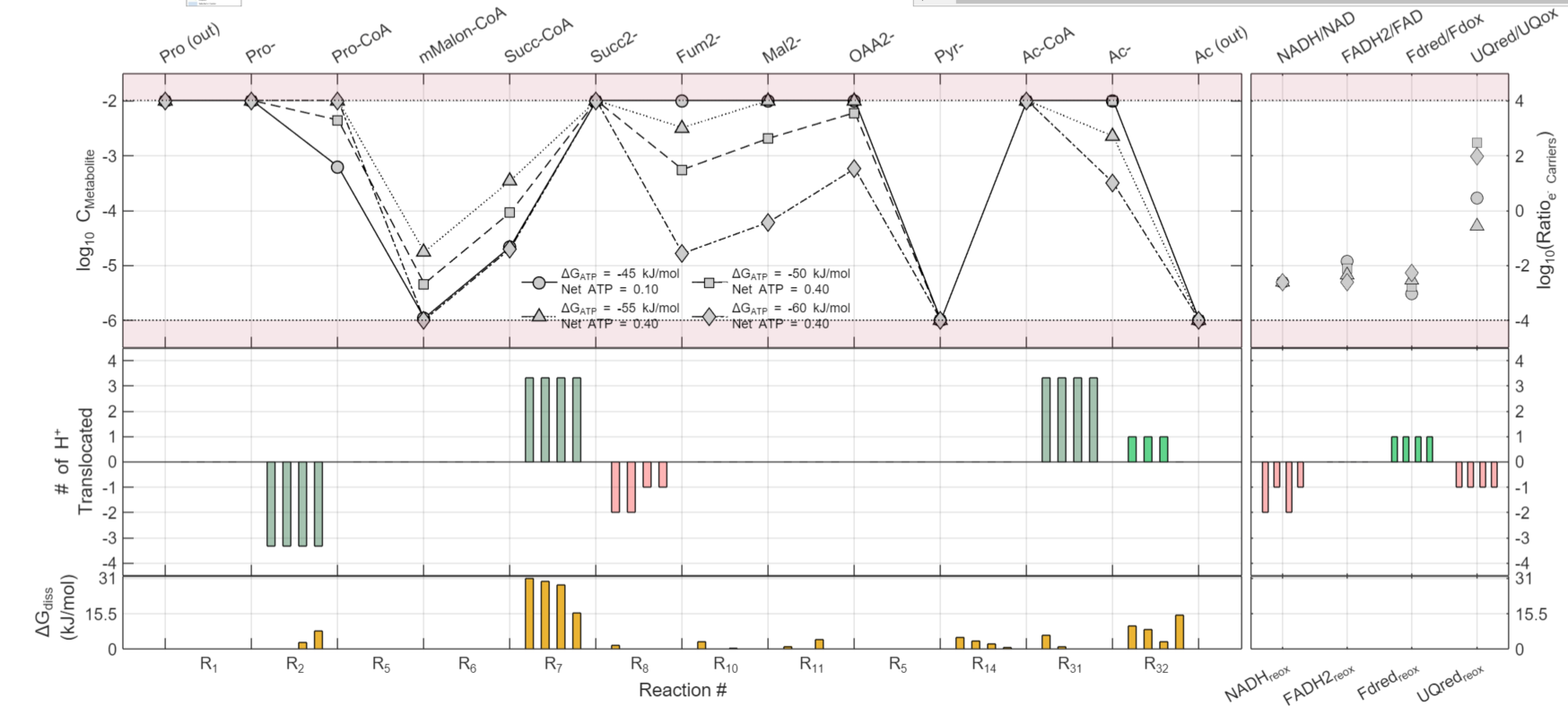

Supplement: FIG S6 [file mSystems.00814-20-sf006.tif]
