# Supplementary material for: Comprehensive Bioenergetic Evaluation of Microbial Pathway Variants in Syntrophic Propionate Oxidation
Source: mSystems. 2020 Dec 8;5(6):e00814-20. doi: 10.1128/mSystems.00814-20 (PMC7743110; doi:10.1128/mSystems.00814-20)
Supplement: TEXT S1 [file mSystems.00814-20-s0001.docx]

Supplementary Material

**Optimal microbial pathway variants can be determined by large-scale bioenergetic evaluation in syntrophic propionate oxidation**

Mauricio Patón^*^, Héctor H. Hernández^**^ and Jorge Rodríguez^*^**^†^**

^*^Department of Chemical Engineering. Khalifa University. Sas Al Nakhl Campus. PO Box 127788 Abu Dhabi, United Arab Emirates.

^**^Department of Biomedical Engineering. Khalifa University. Masdar Campus. PO Box 54224 Abu Dhabi, United Arab Emirates

**^†^** contact: *jorge.rodriguez@ku.ac.ae*

# S1.1. Algorithms used for pathway evaluation

In this section, the algorithms used are described. To find the results of one simulation, please refer to Supplementary Material S3.

## S1.1.1. Algorithm to solve pathways with loop reactions

As described in the manuscript in section *Assessment of pathways feasibility* and in the Supplementary Material Figure S1, some of the pathways (P2, P3, P4 and P6) require the solution of a cyclical pathway. To solve these pathways, a special algorithm was required. A schematic representation of this algorithm is presented in Figure S2.

## S1.1.2. Algorithm to solve branched pathways

As described in the manuscript in section *Assessment of pathways feasibility* and in the Supplementary Material S1, the pathway for *Smithella* (P_7_) requires the solution of a branched pathway. To solve these pathways, a special algorithm was required. A schematic representation of this algorithm is presented in Figure S3.

## S2.3. Algorithm to solve pathways with electron bifurcation

As described in the manuscript in the section *Assessment of pathways feasibility*, the hydrogenotrophic methanogenesis pathway required the solution of an electron bifurcation reaction. In this case, ferredoxin is involved in reactions 32 and 40 (Table 2). In addition, the concentrations of CoB-SH, CoM-S-S-CoB and CoM-SH are unknown. To optimize the energy recovery in the pathway, a special algorithm was developed. A schematic representation of this algorithm is presented in Figure S3.

# S1.2. Enthalpy estimation of CoA-based components

The enthalpies of some of the metabolites were not available in the literature. Those metabolites which enthalpies were not found were the Coenzyme A-activated components.

To overcome this limitation, an estimation of the enthalpies of the metabolites was conducted. The procedure followed consisted in two main steps:

1. *Estimation of the enthalpy in aqueous solution of the Coenzyme A*

Based on the enthalpies of the reactions obtained from literature (1), the change of enthalpy of a compound when it is activated with CoA was estimated as follows:


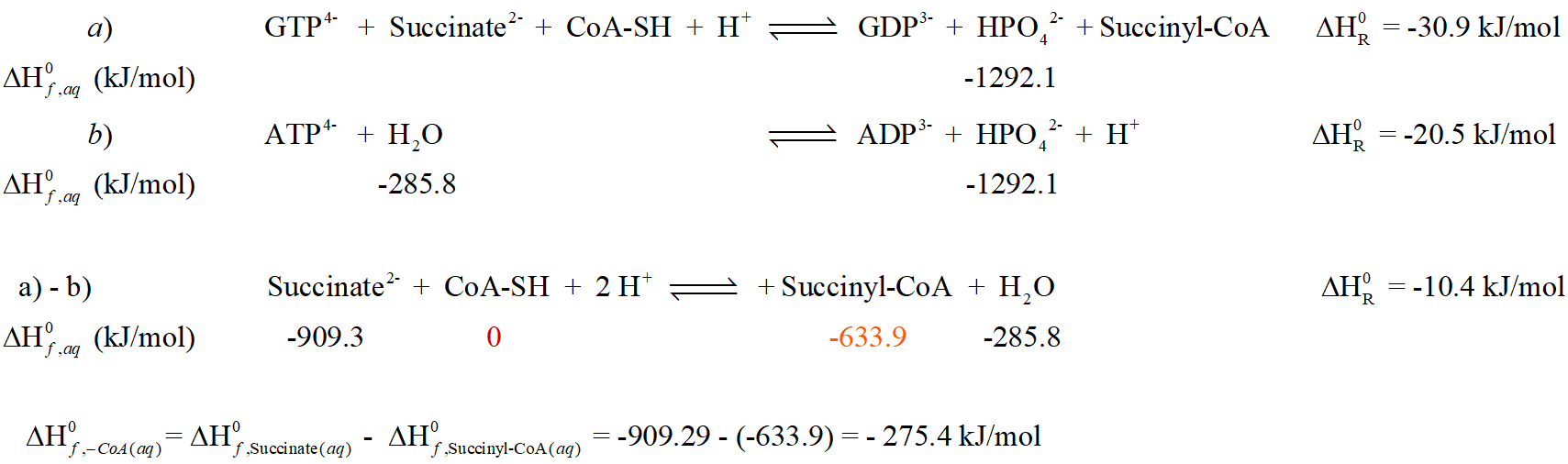


1. *Estimation of the enthalpies of the metabolite without the coenzyme A and add the enthalpy change of CoA.*

Since the enthalpy of the metabolites activated with CoA was unavailable, a search was conducted to find the enthalpies of the inactivated (without CoA) metabolites. The enthalpies of these metabolites however were also not available for aqueous solution. Therefore, an alternative procedure to estimate their ΔH^0^_f_ in aqueous solution was followed.

1. The enthalpies of formation for the inactivated metabolites in gaseous phase of the inactivated metabolites were estimated by using based on enthalpies by group contributions (2):
2. The enthalpies of formation for the inactivated metabolites in liquid phase was estimated by adding the enthalpy of vaporization to the enthalpy of formation in the gas phase.
3. The difference between the enthalpy in the liquid phase between propionic acid and the inactivated metabolite was calculated and added to the enthalpy of the propionic acid in aqueous solution.

For the calculations, please refer to Supplementary Material S2.

# S1.3. Additional figures in support of the discussion of results

In this section, additional figures supporting the results and discussion section are presented for further clarification. The effect of the different parameters are shown for the pathways discussed in the main text.

## S1.3.1. Effect of parameters in the lactate pathway (P4)

Effect of the Ratio H^+^/ATP

In the manuscript, the impact of the ratio H^+^/ATP in the net ATP produced was discussed. The intracellular concentrations of the intermediate metabolites at different H^+^/ATP ratios for the lactate pathway are shown in Figure S4.

In the manuscript, it was described that “the value of the ratio H^+^/ATP (Figure 2b) shows that a small energy quantum (up to an optimum ratio H^+^/ATP of 14/3) could extremely favour the efficiency of the lactate pathway with an almost complete conservation of the entire catabolic energy”. In Figure S5.1, it can be observed that the pathway produces most ATP up to a ratio H^+^/ATP of 14/3. Above this value, the concentration of acryloyl-CoA would fall below the minimum concentration required (1 μM) and therefore, one proton less is translocated.

Effect of intracellular pH

In the manuscript, it was discussed that the net ATP yields by the lactate pathway appear to be unaffected by pH at values above 6.5. The intracellular concentrations of the intermediate metabolites at different intracellular pH values are shown in Figure S5.

The effect of the pH in the pathway at different pH values is shown in the intracellular concentration of propionate. Different intermediate concentrations are observed. However, the net ATP produced under these conditions remains largely unaffected.

*S5.2. Effect of parameters in the methylmalonyl-CoA Pathway (P_2_)*

Effect of ΔG_ATP_

In the manuscript, the impact of the ΔG_ATP_ is discussed for the methylmalonyl-CoA pathway. The intracellular concentrations of the intermediate metabolites at different ΔG_ATP_ are shown in Figure S7:

As seen in Figure S6, in the linear pathway (P1), ΔG_ATP_ values more positive than -60 kJ/mol do not appear to lead to sufficient energy to ultimately drive the endergonic conversion of oxaloacetate to pyruvate. A similar case occurs with the ΔG_ATP_ in the cyclical pathway (Figure S7), where the need to invest one proton less than the other reactions allows for a net positive recovery.

# References

1. Li X, Wu F, Qi F, Beard DA. A database of thermodynamic properties of the reactions of glycolysis, the tricarboxylic acid cycle, and the pentose phosphate pathway. Database [Internet]. ;2011(bar005).

2. Joback KG, Reid RC. Estimation of Pure-Component Properties from Group-Contributions. Chem Eng Commun. 1987 Jul 1;57(1–6):233–43.
