# Supplementary material for: Comprehensive Bioenergetic Evaluation of Microbial Pathway Variants in Syntrophic Propionate Oxidation
Source: mSystems. 2020 Dec 8;5(6):e00814-20. doi: 10.1128/mSystems.00814-20 (PMC7743110; doi:10.1128/mSystems.00814-20)
Supplement: TABLE S1 [file mSystems.00814-20-st001.docx]

Table S1. Name of the metabolites and their thermodynamic properties used. Estimated enthalpy values are shown in blue

| **Full Name** | **Abbreviated Name** | **ΔG^0^_f_ (kJ/mol)** | **ΔH^0^_f_ (kJ/mol)** |
| --- | --- | --- | --- |
| Propionate | Pro- | -361.1 | -510.4 |
| Propionyl CoA | Pro-CoA | -131.3 | -235.0 |
| Acryloyl CoA | Acryl-CoA | -48.5 | -109.0 |
| Lactoyl CoA | Lac-CoA | -287.8 | -411.6 |
| Lactate | Lac- | -517.8 | -687.0 |
| Methylmalonyl CoA | mMalon-CoA | -454.7 | -644.4 |
| Succinyl CoA | Succ-CoA | -461.9 | -633.9 |
| Succinate | Succ2- | -690.9 | -909.3 |
| Fumarate | Fum2- | -602.3 | -777.9 |
| Malate | Mal2- | -843.2 | -1079.8 |
| Oxaloacetate | OAA2- | -793.8 | -959.9 |
| Pyruvate | Pyr- | -472.3 | -596.2 |
| Hydroxypropionyl-CoA | HydroxPro-CoA | -285.0 | -414.0 |
| Hydroxypropionate | HydroxPro- | -518.4 | -689.4 |
| Malonate semialdehyde | Malon Semiald | -477.6 | -814.6 |
| Malonyl-CoA | Mal-CoA | -497.4 | -523.9 |
| Acetyl CoA | Ac-CoA | -140.7 | -210.6 |
| Acetate | Ac- | -369.4 | -486.0 |
| Hydrogen | H2 | 17.6 | -4.2 |
| NAD+ | NAD+ | 0.0 | NA |
| NADH | NADH | 21.8 | NA |
| Carbon Dioxide | CO2 | -386.0 | -413.8 |
| NADP+ | NADP+ | 0.0 | NA |
| NADPH | NADPH | 21.8 | NA |
| FAD | FAD | 0.0 | NA |
| FADH2 | FADH2 | -37.4 | NA |
| Ferredoxin (ox) | Fdox | 0.0 | NA |
| Ferredoxin (red) | Fdred | 79.1 | NA |
| Ubiquinone (ox) | UQox | 0.0 | NA |
| Ubiquinone (red) | UQred | -89.9 | NA |
| Water | H2O | -237.2 | -285.8 |
| Ammonium | NH4+ | -79.4 | -133.3 |
| Coenzyme A | CoA-SH | 0.0 | NA |
| Proton | H+ | 0.0 | 0.0 |
| Methanofuran | MFR | 0.0 | NA |
| Formyl methanofuran | Formyl-MFR | -158.0 | NA |
| Tetrahydromethanopterin | H4MPT | 0.0 | NA |
| Formyl Tetrahydromethanopterin | Formyl-H4MPT | -163.0 | NA |
| Methenyl Tetrahydromethanopterin | Methenyl-H4MPT | 29.3 | NA |
| Methylene Tetrahydromethanopterin | Methylene-H4MPT | 64.7 | NA |
| Methyl Tetrahydromethanopterin | Methyl-H4MPT | 48.2 | NA |
| Coenzyme M | CoM-SH | 0.0 | NA |
| Methyl Coenzyme M | Methyl-CoM | 18.2 | NA |
| Coenzyme B | CoB-SH | 0.0 | NA |
| CoB-CoM heterodisulfide | CoM-S-S-CoB | 39.0 | NA |
